# Poly (ethylene glycol) hydrogel elasticity influences human mesenchymal stem cell behavior

**DOI:** 10.1093/rb/rby008

**Published:** 2018-04-24

**Authors:** Anna K Whitehead, Haley H Barnett, Mary E Caldorera-Moore, Jamie J Newman

**Affiliations:** 1 School of Biological Sciences; 2 Department of Biomedical Engineering, Louisiana Tech University, Ruston, LA 71272, USA

**Keywords:** biomaterial–cell interaction, scaffolds, stem cells

## Abstract

Coordinated investigations into the interactions between biologically mimicking (biomimetic) material constructs and stem cells advance the potential for the regeneration and possible direct replacement of diseased cells and tissues. Any clinically relevant therapies will require the development and optimization of methods that mass produce fully functional cells and tissues. Despite advances in the design and synthesis of biomaterial scaffolds, one of the biggest obstacles facing tissue engineering is understanding how specific extracellular cues produced by biomaterial scaffolds influence the proliferation and differentiation of various cell sources. Matrix elasticity is one such tailorable property of synthetic scaffolds that is known to differ between tissues. Here, we investigate the interactions between an elastically tailorable polyethylene glycol (PEG)-based hydrogel platform and human bone marrow-derived mesenchymal stem cells (hMSCs). For these studies, two different hydrogel compositions with elastic moduli in the ranges of 50–60 kPa and 8–10 kPa were implemented. Our findings demonstrate that the different elasticities in this platform can produce changes in hMSC morphology and proliferation, indicating that the platform can be implemented to produce changes in hMSC behavior and cell state for a broad range of tissue engineering and regenerative applications. Furthermore, we show that the platform’s different elasticities influence stem cell differentiation potential, particularly when promoting stem cell differentiation toward cell types from tissues with stiffer elasticity. These findings add to the evolving and expanding library of information on stem cell–biomaterial interactions and opens the door for continued exploration into PEG-based hydrogel scaffolds for tissue engineering and regenerative medicine applications.

## Introduction

Parallel advances in biologically mimicking (biomimetic) material constructs and in stem cell technologies enable the restoration and direct replacement of diseased cells and tissues. To achieve these outcomes clinically, fully functional cells and tissues must be produced on a large scale [1]. Despite advances in the design and synthesis of biomaterial scaffolds, one of the biggest obstacles facing tissue engineering is a lack of understanding regarding the influence of extracellular cues on cell proliferation and differentiation. The extracellular matrix (ECM) is a highly defined and specialized microenvironment, which is essential for tissue development and function. The ultimate decision of a cell to differentiate, proliferate, migrate, apoptose or perform other functions is a coordinated response to the physical and chemical interactions with these ECM effectors [2].

Matrix elasticity is one mechanical property of the ECM that differs between tissues and can be manipulated in synthesized scaffolds to enhance tissue engineering success and applications [3]. Several studies have demonstrated that matrix elasticity can influence stem cell behavior and differentiation toward certain lineages, indicating the power of physical environment on cell state [4–10]. Notably, Engler et al. initially demonstrated that lineage specification in stem cells can be directed by altering the elastic modulus of polyacrylamide (PA) gels, showing that elasticities of 0.1–1, 8–17 and 25–40 kPa influence mesenchymal stem cell (MSC) differentiation toward neurogenic, myogenic and osteogenic lineages, respectively [6]. Later, Wen et al. systematically modulated the porosity, ligand density and stiffness of PA hydrogels, demonstrating that varying substrate porosity did not significantly change the osteogenic and adipogenic differentiation of human adipose-derived stromal cells and marrow-derived mesenchymal stromal cells. These findings imply that the stiffness of planar matrices regulates stem cell differentiation independently of protein tethering and porosity [11]. Despite the studies mentioned above, there remains a lack of understanding regarding the distinct roles of physical and chemical cues on specific stem cell types. The influence of the dynamic extracellular cues of biologically relevant scaffolds on stem cell proliferation and differentiation remains unclear and further investigation is needed. Understanding these interactions is paramount for the field of tissue engineering and regenerative medicine to more fully advance to clinical applications.

The importance of elasticity in influencing and directing cell behavior generates a need for tailorable biomaterial scaffolds. Hydrogel-based biomaterials have rapidly become an attractive medium because their innate network closely resembles the structure of the ECM, their elasticity can be tailored, they allow for rapid diffusion of hydrophilic nutrients and they have a low content of dry mass, which reduces irritation and degradation [12]. These features allow the hydrogels to provide an environment that is like that of the *in vivo* environment, as well as provide additional control of the physical and mechanical properties affecting cellular proliferation and differentiation. To be effective, the hydrogel scaffold must be capable of promoting desirable cellular functions for specific applications without causing an inflammatory response. Different polymers used to engineer hydrogel scaffolds have different biological properties, all with their own strengths and weaknesses. For example, polyethylene glycol (PEG) polymers are biocompatible and bio-inert in nature. While PEG has been studied for multiple applications, the usefulness of PEG polymers for the formation of tailorable biomimetic scaffolds in tissue engineering and regenerative medicine has not been fully investigated [13–15]. However, PEG acrylates are popular polymers utilized as hydrogel biomaterials for tissue engineering applications [16]. Previously, we demonstrated the generation of biomaterial scaffolds of varying elasticity by implementing tailorable PEG hydrogels. Results showed that our hydrogel platform is compatible with multiple stem cell types, specifically mouse embryonic stem cells, human adipose stem cells and human bone marrow-derived MSCs (hMSCs) [17]. Here, we further characterize the interactions of our hydrogel platform with hMSCs, presenting an investigation into the specific interactions between hMSCs and our tailorable, affordable and reproducible PEG-based hydrogel platform. MSCs are adult, multipotent stem cells harvested from bone marrow, adipose tissue, umbilical cords and muscle [18–31]. MSCs are known for their ability to differentiate into cell types of the mesoderm lineage, with their differentiation into adipogenic, osteogenic and chondrogenic lineages being well described [22, 23]. These cells have the potential to be patient specific and, with several regenerative and immunosuppressive properties, clinically relevant, having been used in approximately 700 clinical trials [24]. MSCs are currently being investigated as potential cell sources to regenerate bone tissue, cartilage, ligament tissue, muscle and adipose tissue [25–29].

To analyze the interactions between bone marrow-derived hMSCs and a hydrogel platform, we selected two hydrogel compositions: 10% wt. PEG dimetharcylate (PEGDMA) MW 1000 and 10% wt. PEGDMA MW 20 000 and the 3% wt. PEGDMA MW 1000 and 17% wt. PEGDMA MW 20 000, which yield elastic moduli in the ranges of 50–60 and 8–10 kPa, respectively. These two hydrogel compositions were chosen because they are at the upper and lower ends of the physiologically relevant elasticities. For conciseness, the hydrogels with an elastic modulus of 50–60 kPa are referred to as ‘stiff hydrogels’ and the hydrogels with an elastic modulus of 8–10 kPa are referred to as ‘soft hydrogels’. Expanding on our previous work, here we demonstrate the utilization of our PEG-based hydrogel blends to study the effect of elasticity on the characteristics and differentiation potential of bone marrow-derived MSCs [17]. We show that the hydrogels of different elasticities produce changes in hMSC morphology and proliferation, which provides support that the platform has the potential to produce changes in hMSC behavior and cell state. Furthermore, we find that the different elasticities can subtly influence stem cell differentiation potential, primarily in cell types of stiffer elasticity. Our findings enhance the fundamental understanding of stem cell–biomaterial interactions and open the door for the continued exploration of PEG-based hydrogel scaffold in tissue engineering and regenerative medicine.

## Materials and methods

### Materials and reagents

#### Hydrogel synthesis and characterization

PEGDMA MW 1000 and MW 20 000 were purchased from Polysciences and were used as received. The ultraviolet (UV) photoinitiator, 2-hydroxy-1-[4-(hydroxyethoxy) phenyl]-2-methyl-1 propanone (I2959) and fibronectin were purchased from Sigma Aldrich. Methacrylate acid (MAA) was purchased from Fisher Scientific and was passed through a basic alumina column prior to use to remove inhibitor. Heptane was purchased from Fisher Scientific. 1-Ethyl-3-(3-dimethylaminopropyl) carbodiimide (EDC), sulfo-N-hydroxysulfosuccinimide (sulfo-NHS), 2-(N-morpholino)ethanesulfonic acid (MES) Buffer, and 1X phosphate buffer saline (PBS) were purchased from Thermo Fisher Scientific.

#### Stem cell maintenance and characterization

Human MSCs were provided by Dr. Bruce Bunnell from Tulane University. Adipogenic differentiation media and osteogenic differentiation media were purchased from LaCell. MEM α, l-Glutamine, Penicillin Streptomycin, ReadyProbes^®^ Cell Viability Imaging Kit (Blue/Red), Alexa Fluor 555 Phalloidin and TRIzol reagent were purchased from ThermoFisher Scientific. Fetal bovine serum was purchased from Atlanta Biologicals. Formalin was purchased from Azer Scientific. Triton X-100 was purchased from Alfa Aesar. Methanol was purchased from VWR. Bovine serum albumin was purchased from Amresco. qScript cDNA SuperMix was purchased from Quanta Biosciences. Powerup SYBR green master mix was purchased from Applied Biosystems. AlamarBlue^®^ reagent and 4',6-diamidino-2-phenylindole, dihydrochloride (DAPI) was purchased from ThermoFisher Scientific.

### Hydrogel preparation

Hydrogel solutions for the ‘stiff’ hydrogels (10% wt. PEGDMA MW 1000 and 10% wt. PEGDMA MW 20 000) and the ‘soft’ hydrogels (3% wt. PEGDMA MW 1000 and 17% wt. PEGDMA MW 20 000) were prepared in deionized water (DH_2_O) as reported previously [30]. 0.1% wt. UV photoinitiator, 2-hydroxyl-1-[4-(hydroxyl) phenyl]-2-methyl-1 propanone (I2959), which is below concentrations previously determined to be cyto-compatible [31], and 2% wt. MAA were added to the hydrogel solution. Solution was sonicated for 20 minutes and then pipetted in between two photomasks separated by 0.55-mm stripes of teflon and UV polymerized at a wavelength of 365 nm and an intensity of ∼34 mW/cm^2^. Stiff and soft hydrogels were UV polymerized for 10 and 20 minutes, respectively. The hydrogels were then rinsed for 10 days in DH_2_O (periodically changed) to remove any un-reacted polymer or monomer. Prior to cell culture, hydrogels were functionalized with fibronectin via EDC/Sulfo-NHS chemistry as described previously [17].

### Characterization of hydrogel swelling

Hydrogel swelling studies were performed as reported previously [29, 32]. After UV polymerization, hydrogel films were cut into ∼19.5-mm discs and were weighed in air as well as in heptane (a solvent the PEG hydrogels will not swell in) to obtain the volume of the hydrogels immediately after UV polymerization. The hydrogels were then rinsed for 10 days in DH_2_O (periodically changed) to remove any un-reacted polymer. Hydrogel discs were then dried for 5 days under vacuum and subsequently weighed to obtain dry (or polymer) mass. The dried hydrogels were then swollen for 48 hours in DH_2_O to reach swollen equilibrium. The polymer volume fraction in the swollen state, *ν*_2,s_ and relaxed state *ν*_2,r_ was calculated from the measured hydrogel mass in air and in heptane:

(1) $$v_{2,s}=\frac{W_{a,d}-W_{n,d}}{W_{a,s}-W_{n,s}}$$

(2) $$v_{2,r}=\frac{W_{a,d}-W_{n,d}}{W_{a,r}-W_{n,r}}$$

where *W*_a,d_ is the hydrogel weight in dry state in air, *W*_n,d_ is the hydrogel weight in dry state in heptane, *W*_a,s_ is the hydrogel weight in swollen state in air, *W*_n,s_ is the hydrogel weight in swollen state in heptane, *W*_a,r_ is the hydrogel weight in the relaxed state in air and *W*_n,r_ is the hydrogel weight in the relaxed state in heptane. The equilibrium volume swelling ratio (*Q*) was calculated by comparing the ratio of the equilibrium swollen volume with the polymer volume at the dry state [32]. Pore sizes were determined using the equation:

(3) $$\xi =v_{2,s}^{-1/3}{\left(\frac{2C_{n}M_{c}}{M_{r}}\right)}^{1/2}l$$

where ξ is the pore size, *ν*_2,s_ is the polymer volume fraction in the swollen state, *C_n_* is Flory characteristic ratio, *M_c_* is the average molecular weight between crosslinks, *M_r_* is the molecular weight of the monomer, and l is the bond-length along the backbone chain. The *M_c_* is found by using the Merrill and Peppas equation:

(4) $$\frac{1}{\bar{M_{c}}}=\frac{2}{\bar{M_{n}}}-\frac{\left(\frac{\overset{-}{\upsilon }}{V_{1}}\right)\left(\ln\left(1-v_{2,s}\right)+v_{2,s}+\chi_{1}v_{2,s}^{2}\right)}{v_{2,r}\left({\left(\frac{v_{2,s}}{v_{2,r}}\right)}^{\frac{1}{3}}-\left(\frac{v_{2,s}}{2v_{2,r}}\right)\right)}$$

where *M_n_* is the number average molecular weight of the uncrosslinked polymer, $\overset{-}{\upsilon }$ is the specific volume of the polymer, is the molar volume of the water, *V*_1_ is the polymer volume fraction in the relaxed state, and $\chi_{1}$ is the polymer–solvent interaction parameter.

### Maintenance of hMSCs

Human MSCs were cultured on 10-cm polystyrene tissue culture dishes in maintenance medium containing MEM α, l-glutamine, penicillin streptomycin and 16.5% FBS. The cells were incubated at 37°C with 5% CO_2_.

### Osteogenic and adipogenic differentiation

Human MSCs were seeded on tissue culture plates and soft hydrogels at a density of 2.0×10^3^ cells/cm^2^ and grown until 80% confluence. Due to decreased proliferation of hMSCs on stiff hydrogels, hMSCs were seeded on these specific gels at 4.0×10^3^ cells/cm^2^ and attached at 80% confluence. The appropriate differentiation media (adipogenic differentiation media or osteogenic differentiation media) was added to the cells in all cases when cells demonstrated 80% confluence. Differentiation media was changed every 72 hours until time point for analysis.

### Cell viability assay

Cell viability was determined using the ReadyProbes^®^ Cell Viability Imaging Kit (Blue/Red) and imaged on the EVOS FL imaging system. Assay was done following manufacturer's protocol.

### F-actin staining

Human MSCs were fixed with formalin and permeabilized using 0.2% Triton 100X. Alexa Fluor 555 Phalloidin was dissolved in methanol to create a stock solution with a final concentration of 200 units/ml. The final staining solution contained a 1:40 ratio of methanolic stock to PBS, with 1% BSA. Cells were protected from direct light and incubated in staining solution for 15 minutes. DAPI was added to each well at a final concentration of 1:2000 and incubated for an additional 5 minutes. Cells were washed with PBS three times and imaged.

### Cell attachment studies

Human MSCs were seeded at a density of 2.0×10^3^ cells/cm^2^ per sample and allowed to attach for 18 hours. Cells were fixed with formalin and permeabilized with 0.2% Triton X-100. Cells were incubated in a 1:1000 solution of DAPI and blocking buffer (0.2% Triton X-100 and 1% wt. BSA in 1X PBS) for 10 minutes. Cells were washed with PBS three times, and 500 µl of PBS was added to each well for imaging. The fluorescence was visualized and imaged using the EVOS FL cell imaging system. Three images were taken per well (top, middle and bottom). ImageJ was used to count the nuclei per image. The average of the three images was taken for each sample.

### Quantitative RT-PCR

RNA was collected and extracted from each cell type using TRIzol reagent following the manufacturer’s protocol. The RNA was quantified using a Take3 plate on a BioTek plate reader. RNA concentrations used for cDNA synthesis are shown in Supplementary Table S1. Due to low RNA concentrations in undifferentiated MSCs, each sample for that experiment was a pool of three wells from a 24-well plate. cDNA was synthesized following the protocol provided by Quanta Biosciences for their cDNA SuperMix kit. The expression levels for each marker were quantified by qRT-PCR according to the manufacturer’s protocol on an Applied Biosystems StepOne Plus instrument (Primer pairs shown in Supplementary Table S2). Each reaction was performed in triplicate for every sample and the relative expression levels were determined by normalizing to *gapdh*.

### AlamarBlue

hMSCs were seeded at a density of 2.0×10^3^ cells/cm^2^ on all three elasticity conditions and grown under standard conditions for 72 hours. At 72 hours, alamarBlue^®^ reagent was added to culture media at 10% of the sample volume. Blanks for each sample were prepared by adding equivalent amounts of culture media and alamarBlue^®^ reagent to wells containing corresponding elasticity conditions, without hMSCs. Samples were incubated at 37°C and protected from direct light. Readings were taken at 1, 2, 3, 4 and 24 hours post alamarBlue^®^ reagent introduction. Fluorescence was measured at excitation 560/emission 590 using a BioTek Cytation 5 plate reader.

### Statistical analysis

All data are expressed as mean with error bars representing Standard Error (SE) for all quantitative comparison experiments. Statistical analysis was carried out via one-way analysis of variance (ANOVA) tests, using SPSS software v 24. *P* < 0.05 was considered statistically significant. Significant results were further analyzed via Tukey Honest Significant Difference (HSD) post hoc test and a *P* values < 0.05 was considered significant.

## Results

### Characterization of hydrogel swelling behavior

Swelling behavior of the synthesized stiff (50–60 kPa) and soft (8–10 kPa) hydrogels was measured to determine the average molecular weight between crosslinks, network pore size and swelling ratio using standard swelling protocols reported previously. The results are summarized in Table 1. While the total percent polymer was held constant at 20% wt. the amount of MW 1000 Da and MW 20 000 Da was varied to create more elastic hydrogels. As expected, the molecular weight between crosslinks and the pore sizes was larger in the soft hydrogels compared to the stiff hydrogels. The equilibrium swelling ratio (Q) of the soft hydrogel formulations is twice that of the stiff hydrogels.

**Table 1:** Pore sizes of stiff and soft hydrogels

	Composition	Elastic modulus (kPa) [23]	*ν*_2,r_	*ν*_2,s_	Mc (g/mol)	Q	ζ (Å)
Stiff hydrogels	10% PEGDMA Mw 20, 000 / 10% PEGDMA Mw 1000	50–60	0.18 ± 5.11E−3	0.094 ± 2.31E−3	1379.11 ± 50.98	10.58 ± 0.26	52.58 ± 1.34
Soft hydrogels	17% PEGDMA Mw 20, 000 / 3% PEGDMA Mw 1000	8–10	0.19 ± 7.71E−3	0.043 ± 1.72E−3	4691.12 ± 130.88	23.45 ± 0.91	127.82 ± 3.21
Patel et al. reference hydrogels	20% PEGDMA Mw 1000	388–390	0.24 ± 0.01	0.18 ± 1.30E−3	256.13 ± 8.32	5.56 ± 0.04	17.77 ± 0.32

### hMSC attachment to hydrogels

**Figure 1:**
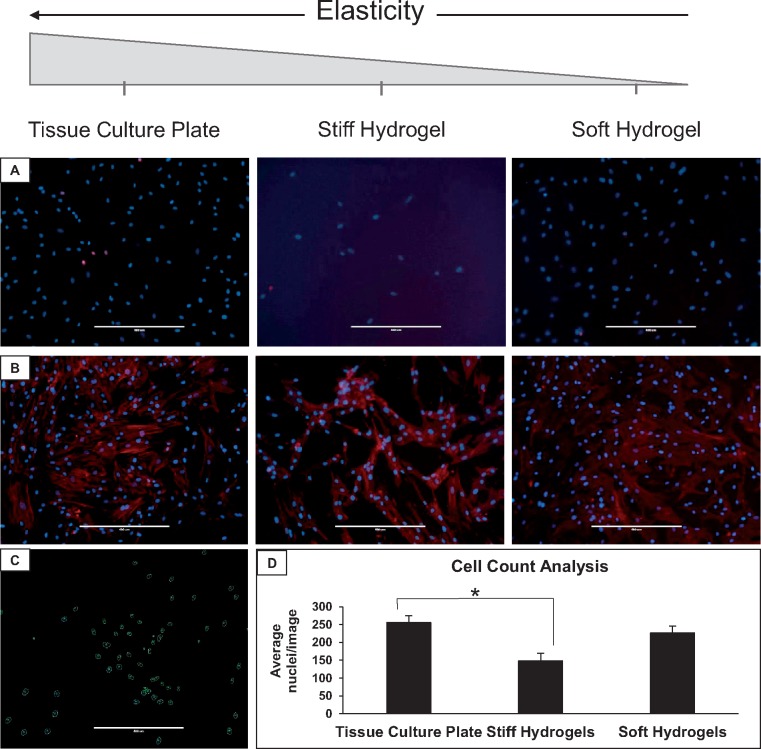
hMSCs attach to and survive on the different hydrogel compositions. (**A**) Viability assay of hMSCs cultured on tissue culture plates, stiff hydrogels and soft hydrogels for 72 hours. Live cell nuclei are shown in blue, while dead cell nuclei are shown in red. (**B**) Morphology of hMSCs cultured on the three elasticity conditions for 72 hours. The cell nuclei are shown in blue, while the F-actin filaments are shown in red. (**C**) Visual depiction of ImageJ analysis highlighting nuclei for count. (**D**) Cell count results from ImageJ quantification of cells seeded for 72 hours. *Tukey HSD resulting *P* < 0.05. *n* = 3. Scale bars: 400 µm

Bone marrow-derived hMSCs were seeded on the hydrogel scaffolds and after 72 hours a viability assay was performed to determine if the cells survived on each of the three surfaces: tissue culture plates, soft hydrogels and stiff hydrogels. Based on propidium iodide staining (dead cells stained red), we observe few, if any, dead cells (Fig. 1A). The difference in image brightness observed from the stiff hydrogels is attributed to the decreased porosity, which further obstructs visualization. The difference in brightness does not alter the number of live/dead cells.

Cell morphology can be an indicator of cellular state, and changes to this morphology could indicate changes in cell behavior. Therefore, F-actin filaments of cells cultured on all three elasticity conditions were stained and visualized (Fig. 1B). Cells on the soft hydrogels maintained similar morphology to the tissue culture plate controls. In contrast, hMSCs cultured on stiff hydrogels displayed a more spindle-like morphology than MSCs cultured on tissue culture plates or soft hydrogels.

ImageJ software was used to analyze the images from the F-actin staining experiment to further confirm differences in cell number observed between the three elasticity conditions. The number of DAPI-stained nuclei in each image was counted and the average of three samples per condition type was determined. Importantly, all hMSCs shown in Fig. 1B were seeded at the same density, cultured for 72 hours and analyzed at the same exposure. As mentioned above, the differences in image brightness observed from the stiff hydrogels is attributed to the decreased porosity, which further obstructs visualization when viewed through an inverted microscope. The difference in brightness does not affect the cell count, as ImageJ was still able to differentiate individual nuclei (Fig. 1C). The cell count analysis revealed a significant difference in the number of nuclei on stiff hydrogels compared to the tissue culture plate control, but no significant difference between the soft hydrogel and that same control was observed (Fig. 1D).

**Figure 2:**
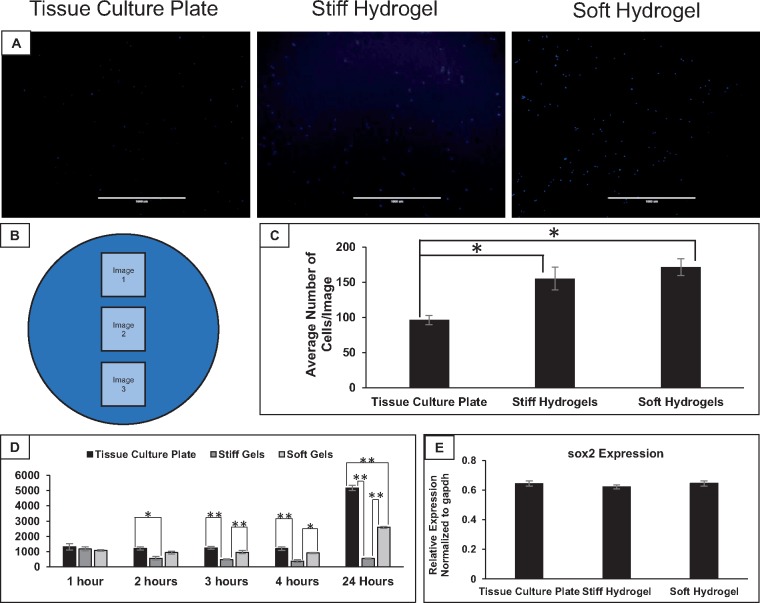
hMSCs attach more readily to hydrogels, but display decreased proliferation, despite equal expression of *sox2*. (**A**) Example images of DAPI stain hMSCs 18 hours post-seeding on the different elasticity conditions. (B) Schematic representation of imaging method used for attachment studies. Three images were taken (as shown in panel A) of each sample, with three samples per surface. (**C**) Results of ImageJ quantification of nuclei per image. (**D**) Results of AlamarBlue analysis of hMSCs cultured on each surface. AlamarBlue was added after cells were cultured for 72 hours, and timepoints shown in graph represent hours after AlamarBlue introduction. (**E**) Quantitative reverse-transcriptase PCR analysis of *sox2* expression in hMSCs cultured on each elasticity condition for 72 hours. *Tukey HSD resulting *P* < 0.05. **Tukey HSD resulting *P* < 0.01. *n* = 3 for C, D and E. Scale bars: 1000 µm

To determine if this difference in cell number was the result of a difference in initial cell attachment, the number of adherent cells was counted 18 hours after seeding. ImageJ analysis of DAPI-stained cells on each surface revealed a significant increase in the number of cells attached to both soft and stiff hydrogels compared to the tissue culture plate control (Fig. 2A–C). This indicates that attachment is not responsible for the decrease in the number of cells present on the hydrogels after 72 hours. Alternatively, differences in rate of proliferation could explain a difference in cell number. An AlamarBlue assay was utilized as an indicator of cellular proliferation, and the results show significantly less metabolic activity in cells cultured on stiff hydrogels compared to soft hydrogels and tissue culture plates at 3, 4 and 24 hours (Fig. 2D). At 24 hours, metabolic activity was significantly higher in cells cultured on tissue culture plates than in cells cultured on both stiff and soft hydrogels. Given that proliferation is slower on the stiff hydrogels, the expression of the multipotency marker *sox2* was analyzed to see if there were significant changes in multipotency. Cells were seeded at the same density on each surface and cultured for 72 hours before collecting RNA. Results of qRT-PCR of *sox2* (Fig. 2E) indicates that there is no statistically significant difference in expression levels between each surface, demonstrating that the elasticity conditions do not immediately influence the levels of certain multipotency transcription factors.

### Effect of elasticity on hMSC osteogenic differentiation

**Figure 3:**
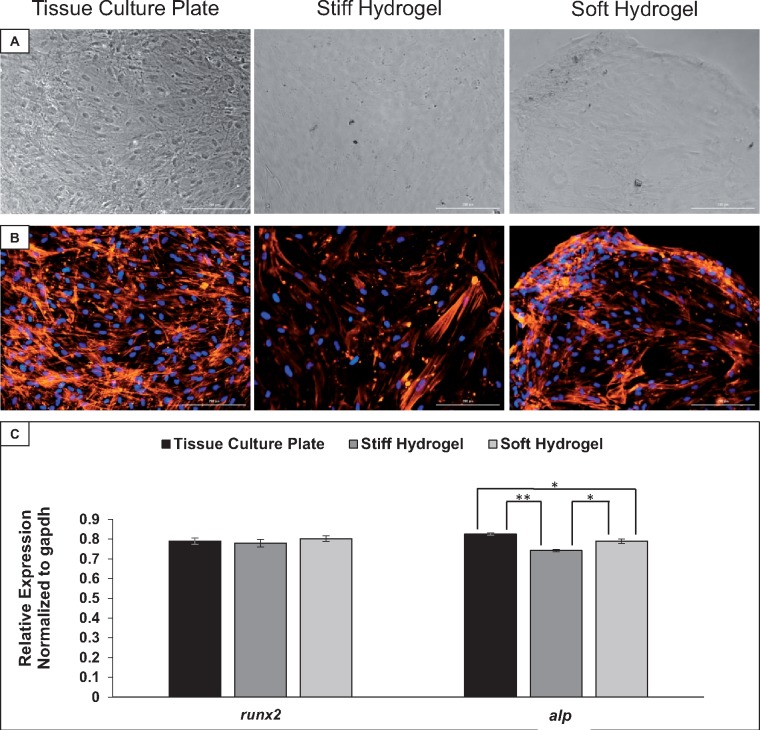
hMSCs retain the ability to differentiate toward osteogenic lineages on all three elasticity conditions. (**A**) Phase contrast images of hMSCs at day 7 of osteogenic differentiation. (**B**) Morphology of hMSCs at day 7 of osteogenic differentiation, corresponding to the phase contrast images in panel A. The cell nuclei are shown in blue, while the F-actin filaments are shown in orange. (**C**) Quantitative reverse-transcriptase PCR analysis of the osteogenic differentiation markers *runx2* and *alp* in hMSCs at day 7 of osteogenic differentiation. *Tukey HSD resulting *P* < 0.05. **Tukey HSD resulting *P* < 0.01. *n* = 3. Scale bars: 200 µm

To be useful in tissue engineering and regenerative medicine, biomaterial scaffolds must be able to support and potentially direct stem cell differentiation toward desired lineages. Elasticity can play a role in directing stem cell state, thus the effects of the hydrogel elasticities on hMSC differentiation toward an osteogenic lineage were investigated. Osteogenic differentiation was chemically induced in hMSCs seeded on all three elasticity conditions, and morphology was analyzed using phase contrast microscopy (Fig. 3A). Due to the limited visibility in phase contrast images with hydrogels, phalloidin staining was also used to visualize F-actin filaments (Fig. 3B). There was noticeable differentiation and calcium deposition on all three elasticity conditions. qRT-PCR of osteogenic markers *runx2* and *alp* (Fig. 3C) was performed on samples collected at day 7 of differentiation. Analysis indicated no significant differences in the early osteogenic differentiation marker *runx2* expression in hMSCs cultured on each surface. However, there were significant differences in *alp* expression, an early marker of osteogenesis, between soft hydrogels and stiff hydrogels (*P* < 0.05), between soft hydrogels and tissue culture plates (*P* < 0.05) and between stiff hydrogels and tissue culture plates (*P* < 0.01).

### Effect of elasticity on hMSC adipogenic differentiation

**Figure 4:**
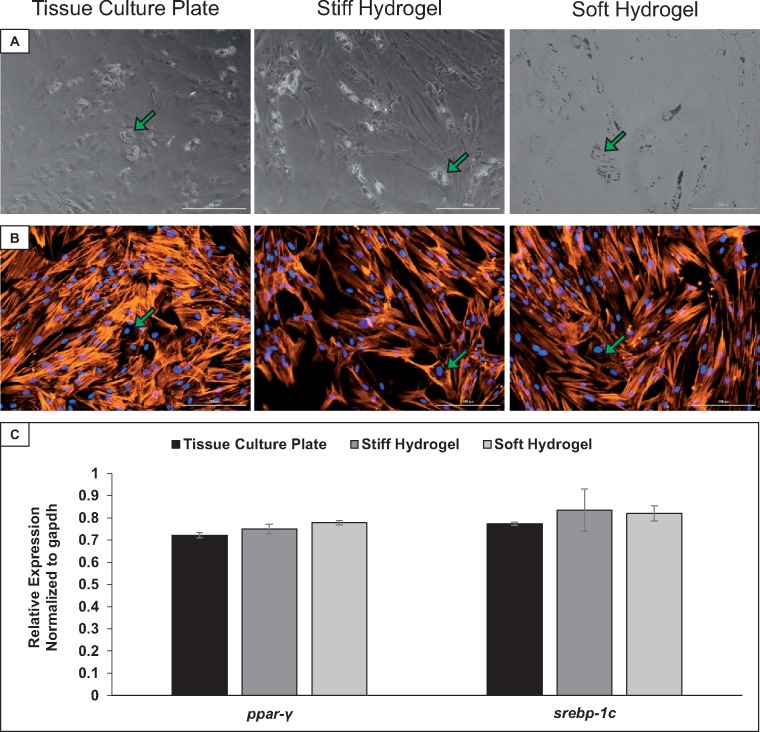
hMSCs retain the ability to differentiate toward adipogenic lineages on all three elasticity conditions. (**A**) Phase contrast images of hMSCs at day 7 of adipogenic differentiation. (**B**) Morphology of hMSCs at day 7 of adipogenic differentiation, corresponding to the phase contrast images in panel A. The cell nuclei are shown in blue, while the F-actin filaments are shown in orange. Cells containing lipid vesicles demonstrated a rearrangement of F-actin filaments, indicated by green arrows. (**C**) quantitative reverse-transcriptase PCR analysis of the adipogenic differentiation markers *ppar-γ* and *srebp1-c* in hMSCs at day 7 of adipogenic differentiation. Results considered insignificant with *P* > 0.05. *n* = 3. Scalebars: 200 µm

Since hMSCs also have the potential to be used for adipogenic tissue regeneration, we further assessed adipogenesis of these cells on each of the selected surfaces. Adipogenic differentiation was chemically induced in cells seeded on all three elasticity conditions, and morphology was analyzed using phase contrast microscopy (Fig. 4A). As in the previous set of experiments, phalloidin staining was used to visualize F-actin filaments and provide higher resolution images of cell morphology (Fig. 4B). Noticeable differentiation had taken place on each surface, with round globules, some of which are indicated by green arrows in Fig. 4A and B, indicating vacuoles and adipogenic differentiation. Phalloidin staining of the cells shows that in areas where lipid vacuoles formed there is a decrease in F-actin filaments. This trend is seen on tissue culture plates, soft hydrogels and stiff hydrogels. qRT-PCR of early adipogenic markers *ppar-y* and *srebp1c* was performed on samples collected at day 7 of differentiation. Analysis indicates no significant differences in expression of these early adipogenic markers between each surface (Fig. 4C).

## Discussion

Previously, we demonstrated that PEGDMA hydrogels with elasticities within a physiologically relevant range (8–60 kPa) can be generated by varying the molecular weight of the polymer [17]. Here, we characterized hydrogels at the upper and lower ends of this range, specifically in terms of their swelling behavior and interactions with hMSCs. The swelling behavior of the hydrogels was used to determine the molecular weight between crosslinks (*M_c_)*, the pore sizes (ξ) and the equilibrium swelling ratio (Q). All three, as predicted, were lower in the stiff hydrogels than in the soft hydrogels. Variability in the swelling characteristics between the two samples was attributed to the higher percentage of PEGDMA MW 20 000 within the soft hydrogels (Table 1). Next, we characterized hMSC interactions when cultured on the stiff and soft hydrogels. As the largest pores are nanometers in size and hMSCs have an approximate diameter range of 17.9–30.4 μm, there is no penetration of hMSCs into the hydrogel network. Thus, hMSCs are cultured two-dimensionally on the surface of these hydrogels.

When seeded on both soft and stiff hydrogels, hMSCs were shown to attach and remain viable (Fig. 1A), further confirming the potential of this platform for use in cell culture and tissue generation. However, changes in morphology were observed in cells cultured on the different elasticities. Human MSCs cultured on the soft hydrogels maintained similar morphology to the tissue culture plate controls. In contrast, hMSCs cultured on stiff hydrogels displayed a more spindle-like morphology as compared to hMSCs cultured on tissue culture plates or soft hydrogels (Fig. 1B). These differences in morphology could indicate a change in cell behavior, such as spontaneous differentiation. Furthermore, there appeared to be consistently fewer hMSCs on the stiff hydrogels after 72 hours of culture. ImageJ quantification of the hMSCs shown in Fig. 1B revealed significantly fewer cells on the stiff hydrogels (Fig. 1D). The decrease in hMSCs could be the result of decreased attachment to the stiff hydrogels. However, attachment analysis 18 hours after seeding actually revealed an increased number of hMSCs attached to both hydrogel elasticities compared to tissue culture plate indicating that the hydrogels have an impact on cell proliferation (Fig. 2A–C).

AlamarBlue assays demonstrated that proliferation is significantly decreased in the cells cultured on the soft and stiff hydrogels. Human MSC proliferation on stiff hydrogels was shown to be significantly decreased 3-hours after the introduction of AlamarBlue (Fig. 2D). The differences in morphology and proliferation observed in hMSCs cultured on stiff hydrogels could be an indication of spontaneous differentiation. Quantitative Reverse-Transcriptase – polymerase chain reaction (RT-PCR) analysis of the multipotency marker *sox2* revealed no significant differences in expression across the three elasticity conditions (Fig. 2E). However, further analysis of multipotency markers and markers of possible differentiation lineages could reveal that a subtle amount of spontaneous differentiation has taken place or longer time course studies may demonstrate more significant changes in multipotency. For the scope of this study, the differentiation potential of hMSCs on the three elasticity conditions was analyzed through chemically induced differentiation toward osteogenic and adipogenic lineages, rather than exploring the long-term effects of maintenance on each of these surfaces.

On all three elasticity conditions, hMSCs cultured in osteogenic differentiation media differentiated toward the osteogenic lineage, as evidenced by calcium deposition and expression of bone specific markers. There was no significant difference in *runx2* expression, which is an essential transcription factor for osteoblastic differentiation (Fig. 3C). However, there was a significant decrease in *alp* expression in hMSCs cultured on both hydrogel elasticities (Fig. 3C). While lower levels of *alp* expression could indicate decreased osteogenesis, given the observation of calcium deposition and cell morphology, it is also possible that a decrease in *alp* expression is an indication of more rapid maturation of the resulting cells.

hMSCs cultured in adipogenic differentiation media also retained the ability to differentiate toward adipogenic lineages on all three elasticity conditions. hMSCs on all three elasticity conditions began forming lipid vesicles characteristic of adipogenic differentiation (Fig. 4A and B). There was no significant difference in the expression of two key transcription factors involved in adipogenesis, *ppar-γ* and *srebp1-c*. Ultimately, no difference in hMSC differentiation toward adipogenic lineages was observed.

In summary, the data from this study give insight into the properties and stem cell interactions of our previously established hydrogel platform; an inexpensive, highly tailorable platform that can be adapted to any number of cell-material interaction studies and applications. There is a need within the field to investigate the roles of both the physical and chemical properties of different biomaterials on a variety of stem cells. This article presents a focused investigation into the interactions between hMSCs and our specific PEG-based hydrogel platform. Changes in hMSC morphology and proliferation were observed in cells cultured on hydrogels, primarily those cultured on stiff hydrogels. These results demonstrate that the elastic tailorability of this hydrogel platform can produce changes in hMSC behavior and cell state, indicating a potential for these hydrogels to be used to generate a controlled environment for cell culture and tissue regeneration applications. Furthermore, based on the differentiation studies, the different hydrogel elasticities have subtle effects on stem cell differentiation. This effect is observed primarily in osteogenic differentiation, which could indicate that cell lineages of higher elasticity are more susceptible to elasticity changes. The results of this study further suggest that the hydrogel platform’s elasticity does affect stem cell behavior, opening the door to future investigations of the platform’s potential for controlling stem cell fate. While the platform is limited in terms of its ability to be tailored for responsiveness and degradation, the limitations are appropriate for current and future studies focused on elasticity and other specific cues, while maintaining affordable and reproducible biomaterial scaffolds. In addition, PEG is an US Food and Drug Administration (FDA)-approved polymer, which gives the platform the potential to be used in the clinic, should future studies open the door for therapeutic uses of these hydrogels. The focus of this study was to understand cell–biomaterial interactions, with the goal of adding to a library of materials for various applications in regenerative medicine. A better understanding of the biomaterial scaffolds utilized in tissue regeneration, such as the studies shown here, is essential in optimizing their translational and clinical potential. 

## Supplementary Material

Supplementary Figure

Supplementary Tables
