# Supplementary figures and images for: Poly (ethylene glycol) hydrogel elasticity influences human mesenchymal stem cell behavior
Source: Regen Biomater. 2018 Apr 24;5(3):167–75. doi: 10.1093/rb/rby008 (PMC6007362; doi:10.1093/rb/rby008)

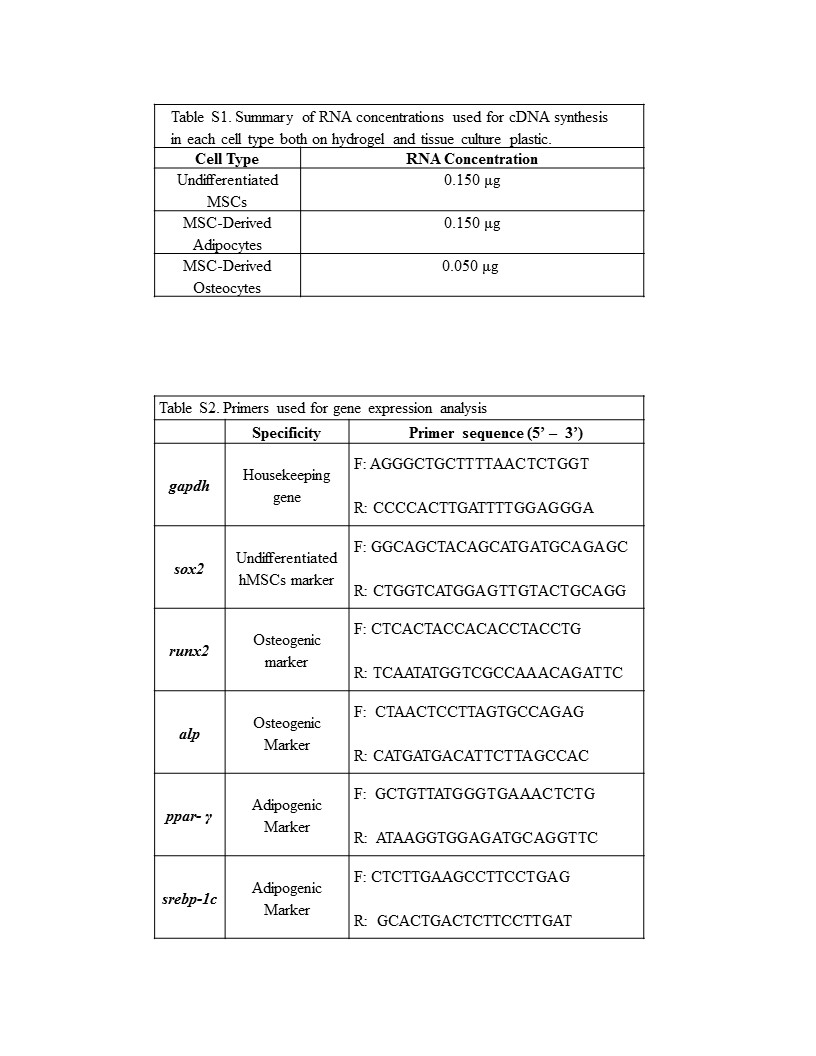

Supplement: Supplementary Figure [file rby008_figure_supplemental.jpeg]
