# Supplementary material for: Poly (ethylene glycol) hydrogel elasticity influences human mesenchymal stem cell behavior
Source: Regen Biomater. 2018 Apr 24;5(3):167–75. doi: 10.1093/rb/rby008 (PMC6007362; doi:10.1093/rb/rby008)
Supplement: Supplementary Tables [file rby008_suppl_tables.pdf]

Table S1. Summary of RNA concentrations used for cDNA synthesis in each cell type both on hydrogel and tissue culture plastic.	
**Cell Type**	**RNA Concentration**
Undifferentiated MSCs	0.150 µg
MSC-Derived Adipocytes	0.150 µg
MSC-Derived Osteocytes	0.050 µg

Table S2. Primers used for gene expression analysis		
	**Specificity**	**Primer sequence (5' – 3')**
gapdh	Housekeeping gene	F: AGGGCTGCTTTTAACTCTGGT<br>R: CCCCACTTGATTTTGGAGGGA
sox2	Undifferentiated hMSCs marker	F: GGCAGCTACAGCATGATGCAGAGC<br>R: CTGGTCATGGAGTTGTACTGCAGG
runx2	Osteogenic marker	F: CTCACTACCACACCTACCTG<br>R: TCAATATGGTCGCCAAACAGATTC
alp	Osteogenic Marker	F: CTAACTCCTTAGTGCCAGAG<br>R: CATGATGACATTCTTAGCCAC
ppar- γ	Adipogenic Marker	F: GCTGTTATGGGTGAAACTCTG<br>R: ATAAGGTGGAGATGCAGGTTC
srebp-1c	Adipogenic Marker	F: CTCTTGAAGCCTTCCTGAG<br>R: GCACTGACTCTTCCTTGAT
